# Characteristics of clinical manifestations and molecular genetics of inherited hyperhomocysteinemia in children and adolescents: a single center experience from China

**DOI:** 10.3389/fneur.2025.1588273

**Published:** 2025-09-09

**Authors:** Rui Qu, Aimin Han, Yang Zhao, Xiangju Qu, Yali Zhu

**Affiliations:** ^1^Department of Pediatrics, The Affiliated Hospital of Xuzhou Medical University, Xuzhou, China; ^2^Department of Ophthalmology, Xuzhou Cancer Hospital, Xuzhou, China; ^3^School of Mechanical and Electrical Engineering, Xuzhou University of Technology, Xuzhou, China

**Keywords:** hyperhomocysteinemia, cystathionine β-synthase, cerebral venous sinus thrombosis, methylenetetrahydrofolate reductase, cobalamin C

## Abstract

**Introduction:**

Accurate identification of the genetic cause of inherited hyperhomocysteinemia (HHcy) is essential for targeted therapies and individualized treatment. However, reported cases in China remain limited. In this study, we investigated the clinical and molecular genetic characteristics of HHcy in Chinese children/adolescents.

**Methods:**

Between 2021 and 2024, eight children/adolescents with inherited HHcy were identified at a tertiary hospital. The patients' clinical presentations, biochemical findings, and genetic profiles were analyzed.

**Results:**

Eight Chinese patients exhibited elevated plasma total homocysteine (tHcy) levels (85.7–227.2 μmol/L). These patients revealed 11 variants across 3 genes, including 2 novel variants and 9 previously reported pathogenic variants. All patients were compound heterozygotes. Six patients (P1–6) were diagnosed with cystathionine β-synthase (CBS) deficiency, with seven *CBS* variants identified. Among these, one novel frameshift variant (c.860del) was detected. Major clinical manifestations included marfanoid features, lens dislocation, myopia, mild developmental delay, osteoporosis, epilepsy, and cerebral venous sinus thrombosis, with ectopia lentis or myopia as common early signs (ages 4–6 years). One child had methylenetetrahydrofolate reductase deficiency, with two variants (c.1632+2T>G, c.1552C>T) and the variant c.1552C>T was novel. The patient displayed developmental delays, microcephaly, and status epilepticus. One child (P8) showed elevated tHHcy and urine methylmalonic acid levels, attributed to cobalamin C deficiency caused by *MMACHC* variants (c.482G>A, c.609G>A). He presented with epilepsy, weakness in both lower limbs, cognitive dysfunction, and urinary incontinence. Comprehensive interventions including dietary and pharmacological therapies, significantly reduced tHHcy levels in most cases.

**Discussion:**

Elevated tHcy is an important biomarker for inherited HHcy. Genetic testing is crucial for precise diagnosis, therapy initiation, and genetic counseling. Two novel pathogenic variants were identified, enriching the variant spectrum for inherited HHcy.

## 1 Introduction

Inherited hyperhomocysteinemia (HHcy) is a treatable inherited genetic disorder characterized by the body's inability to metabolize homocysteine, an amino acid central to various metabolic processes (1). HHcy can result from cystathionine β-synthase (CBS) deficiency, methylenetetrahydrofolate reductase (MTHFR) deficiency, methionine (Met) adenosyl transferase deficiency, or cobalamin metabolic disorders (2, 3). According to plasma total homocysteine (tHcy) concentrations, HHcy is classified as mild (15–30 μmol/L), moderate (31–100 μmol/L), or severe (>100 μmol/L) types (3, 4). This metabolic disorder affects multiple organs, and its clinical, biochemical, and genetic manifestations vary significantly depending on the underlying etiology (5, 6). Many patients remain undiagnosed until severe clinical events occur later in life, with some never receiving a diagnosis (7). Without treatment, inherited HHcy leads to chronic, progressive multisystemic diseases that manifest in early childhood (8). Accurate identification of the genetic cause is essential for targeted therapies and individualized treatment (4). However, despite being included in the first batch of rare disease catalogs in China, reported HHcy cases in China remain limited. This study aims to describe the clinical and biochemical features, variant spectrum, treatment regimens, and follow-up outcomes of eight Chinese children/adolescents with inherited HHcy.

## 2 Materials and methods

In this retrospective study, we analyzed the data of eight children and adolescents with HHcy of proven genetic etiology from six Chinese families who visited the Affiliated Hospital of Xuzhou Medical University between January 2021 and September 2024. All patients had ≥3 months of follow-up data for analysis. All parents were non-consanguineous, and two sibling pairs were identified (P1 and P2, P3 and P4). The other four cases (P5–P8) were unrelated. Six patients (P1, P3, P5–P8) visited our hospital because of cerebral venous sinus thrombosis (CVST), ectopia lentis, status epilepticus (SE), cognitive dysfunction, and urinary incontinence (Table 1). Patients P2 and P4 were identified through family screening. All inherited HHcy cases were diagnosed using biochemical assays (plasma and urinary tHcy, serum Met determination, etc.) and genetic analyses (whole-exome sequencing and Sanger sequencing for genetic variants). Proband DNA samples were subjected to second-generation sequencing using a targeted capture strategy. The pathogenicity of the suspected variants was preliminarily analyzed and identified. Sanger sequencing was performed to verify the presence of loci. Finally, family screening was performed using Sanger sequencing for all patients and their affected siblings.

**Table 1:** Clinical and biochemical features of eight Chinese patients with inherited HHcy.

**Patient/sex**	**Onset**		**Diagnosis**		**Other symptoms**	**Plasma tHcy at diagnosis (μmol/L)**	**Plasma Met at diagnosis (μmol/L)**	**Urine MMA at diagnosis (mg/g Cr)**
	**Age, years**	**Symptoms**	**Age, years**	**Symptoms**				
Pt1^a^/F	4	Myopia	12	CVST	Epilepsy, lens ectopia, MF, mild ID, scoliosis of the spine, OP	119.6	387	N
Pt2^a^/M	5	Myopia	6	Family screening	Inferior lens subluxation, MF, mild ID	104.7	342	N
Pt3^b^/M	4	Lens ectopia	13	CVST	Anemia, epilepsy, mild ID, myopia	227.2	275	N
Pt4^b^/F	4	Myopia	7	Family screening	Lens ectopia, mild ID	118.4	314	N
Pt5/F	5	Myopia	13	CVST	Lens ectopia, MF, mild ID, OP	169.3	438	N
Pt6/M	6	Unclear vision	8	Severe myopia	Lens ectopia	142.3	176	N
Pt7/F	1	Developmental delay	2	SE	Microcephaly	85.7	6.8	N
Pt8/M	6	Unstable walking, epilepsy	12	Cognitive dysfunction, urinary incontinence	Weakness of both lower limbs	103.8	4.5	112.6

^a, b^Siblings.

Pt, patient; F, female; M, male; CVST, cerebral venous sinus thrombosis; ID, intellectual disability; MF, marfanoid features; MMA, methylmalonic acid; OP, osteopenia; SE, status epilepticus; tHcy, total homocysteine; HHcy, hyperhomocysteinemia.

If HHcy with CBS deficiency was determined, pyridoxine responsiveness was further determined by measuring plasma Hcy levels after oral administration of vitamin B6 (300–600 mg/day) for at least 2 weeks. Pyridoxine responders were defined as those with a decrease in plasma Hcy levels to below 50 mmol/L, and patients with little or no decrease were defined as non-responsive. For pyridoxine non-responsive patients, betaine supplementation (3–6 g/day) and a low Met diet was advised (9, 10). All children underwent regular examinations and follow-ups, and professional doctors adjusted their medications according to their condition. The institutional review board approved the study for the study center (XYFY2020-KL093-01), and informed consent was obtained from the parents of all patients.

## 3 Results

### 3.1 Demographic and baseline characteristics

Eight children/adolescents (four females and four males) representing six families were studied, including two sibling pairs (P1/P2 and P3/P4). All parents were healthy, non-consanguineous couples, and all children were born at full term by natural delivery. Symptoms first appeared from infancy to early childhood, with a mean diagnostic age of 9.1 ± 4.0 years (median, 10.0 years; range, 2.0–13.0 years; IQR, 6.3–12.8 years). Four (50.0%) were male. Six patients (P1–P6) were diagnosed with CBS deficiency, one child (P7) with MTHFR deficiency, and one (P8) with cobalamin C (cblC) deficiency. Pyridoxine responsiveness in the CBS deficiency group showed no responders.

### 3.2 Clinical and biochemical characteristics

**Figure 1:**
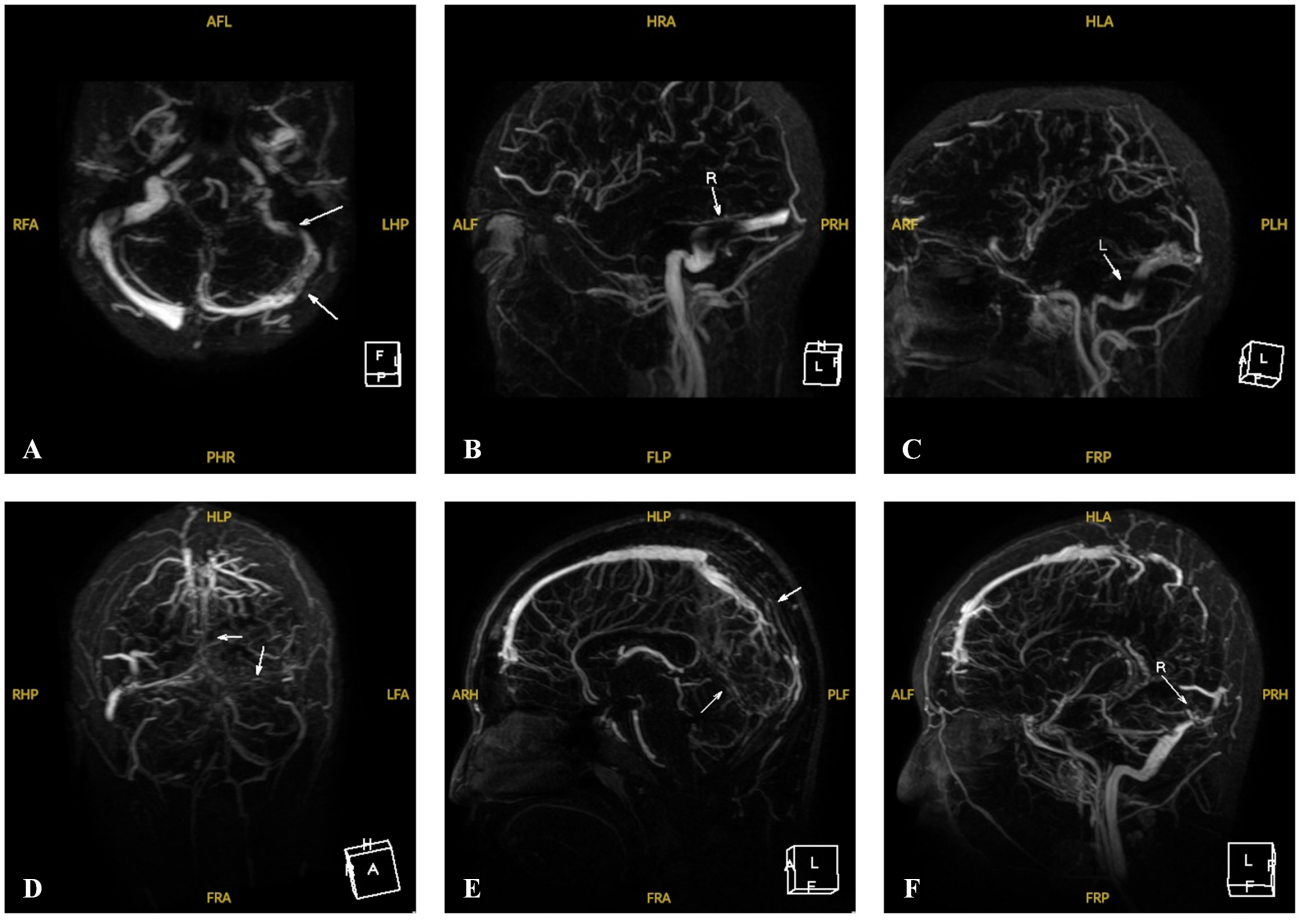
MRV images of Patients 1 and 5 with CVST at the time of onset. **(A–C)** MRV of P1 revealed filling defects involving the left sigmoid sinus, the transverse sinus and its branches at the onset (white arrows). **(D–F)** MRV of P5 revealed multiple filling defects involving the upper sagittal sinus, the sinus confluence and straight sinus, the bilateral transverse sinus, the left sigmoid sinus and the internal jugular vein running area at the onset (white arrows).

The clinical and biochemical features of the eight patients are summarized in Table 1. All patients had significantly elevated plasma tHcy level. The mean plasma tHcy level was 133.9 ± 45.6 μmol/L (median, 119.0 μmol/L; range, 85.7–227.2 μmol/L; IQR, 104.0–162.6 μmol/L). Six patients (P1–P6) were diagnosed with CBS deficiency at ages ranging from 6 to 13 years. The major clinical manifestations of the six patients included marfanoid features, lens dislocation, myopia, mild developmental delay, osteoporosis, CVST, and epilepsy. Eye disorders were universal, with ectopia lentis or myopia being the initial presentation (ages 4–6 years). The mean age at diagnosis for these six patients was 9.8 ± 3.2 years (median, 10.0 years; range, 6.0–13.0 years; IQR, 6.8–13.0 years). The diagnostic symptom in one child (P6) was lens ectopia, with no symptoms typical of a CBS deficiency other than eye disorders. The patient (P6) underwent lensectomy with intraocular lens implantation. The other five patients (P1–P5) had multisystem damage, with CVST being the most common diagnostic symptom (P1, P3, and P5). Three patients (P1, P3, and P5) were hospitalized due to progressively exacerbating severe headaches accompanied by confusion. Magnetic resonance venography (MRV) was performed in two patients (P1 and P5), with findings such as thrombosis in the left transverse sinus, sigmoid sinus, superior sagittal sinus, sinus confluence, straight sinus, bilateral transverse sinus, left sigmoid sinus, and internal jugular vein. Both these neuroimaging findings strongly suggested CVST (Figure 1).

**Figure 2:**
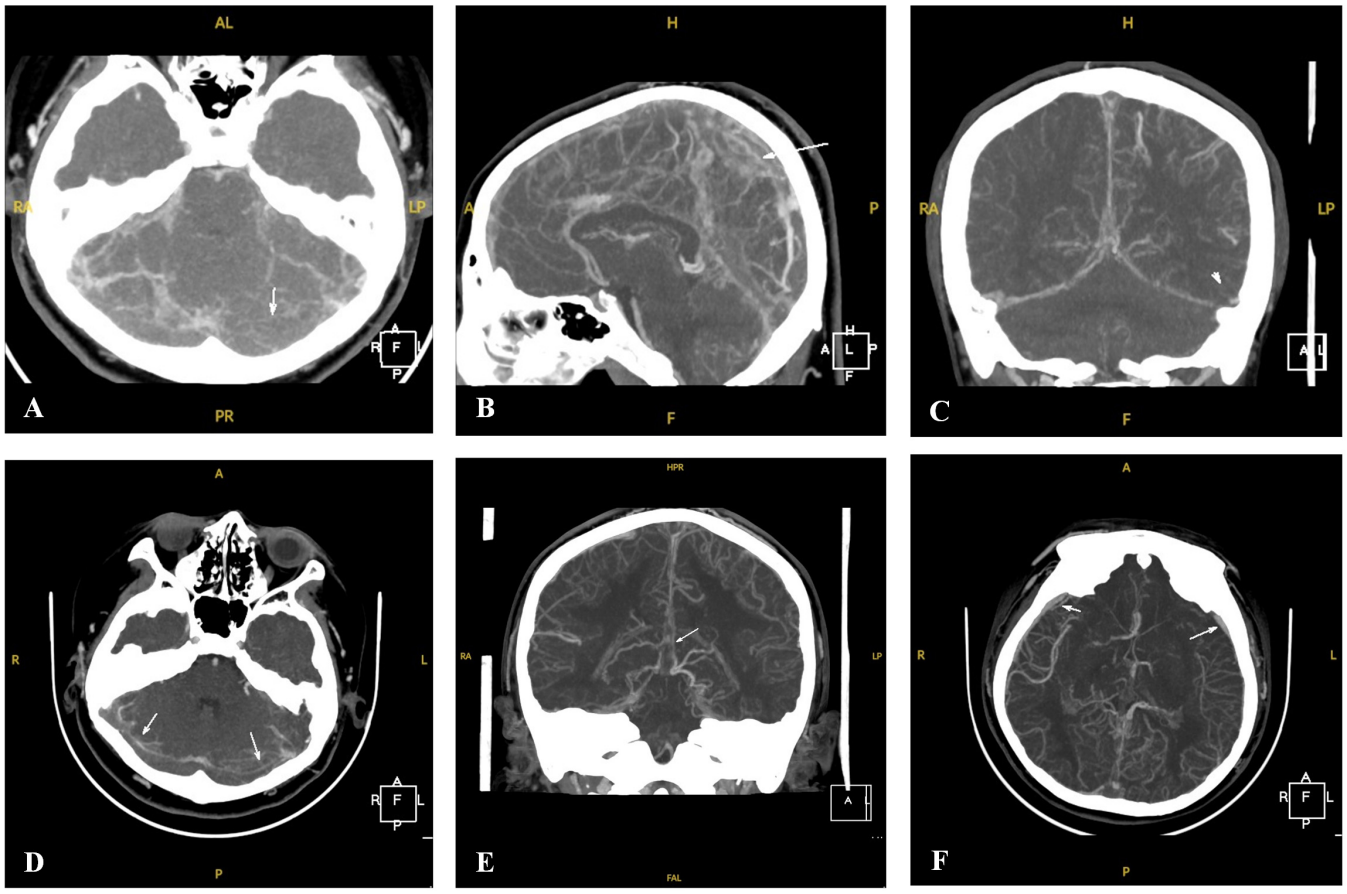
CTV images of Patient 3 with CVST at the time of onset and after percutaneous intracranial venous thrombectomy. **(A–C)** CTV of P3 revealed thin lumens and suspected multiple filling defects involving the superior and inferior sagittal sinuses, straight sinuses, bilateral transverse sinuses, and sigmoid sinuses at the onset (white arrows). **(D–F)** CTV of P3 revealed thin and partially visible lumens and multiple filling defects involving the superior and inferior sagittal sinuses, straight sinuses, bilateral transverse sinuses, and sigmoid sinuses after percutaneous intracranial venous thrombectomy. Multiple thickened tortuous vascular shadows can also be observed in the intracranial and subcutaneous regions (white arrows).

Two patients (P1 and P5) required pediatric intensive care unit admission and were treated with low-molecular-weight heparin (100 U/kg subcutaneously twice daily) for 14 days, followed by an oral anticoagulant (warfarin) to maintain an international normalized ratio of 2–3. Intravenous mannitol and fructose glycerol were administered for elevated intracranial pressure, while levetiracetam was used for seizures. One patient (P3) had extensive venous thrombosis, developed status epilepticus after hospitalization, and underwent percutaneous intracranial venous thrombectomy (Figure 2).

The patient returned to the pediatric intensive care unit and received treatment, including intracranial hypertension management, anticoagulants, and antiepileptics. At a median follow-up of 13 months, the patient showed greatly improved venous sinus thrombosis on cranial MRV, with no relapses. One year later, three patients underwent another 24-h video electroencephalography (VEEG) examination. One child (P5) exhibited a normal VEEG, while two children (P1, P3) exhibited abnormal VEEG results. P1 exhibited discharges in the bilateral occipital and middle posterior temporal regions, while P3 exhibited multifocal and extensive discharges. We continued to administer levetiracetam (P1) and levetiracetam combined with sodium valproate (P3) for antiepileptic treatment. All six children (P1–P6) with CBS deficiency had significantly elevated plasma tHcy (104.7–227.2 μmol/L vs. the normal range of 0–15 μmol/L) and Met levels (176–438 μmol/L vs. the normal range of 8–50 μmol/L). Urine organic acid screening of all six patients showed no abnormalities. As all six patients were pyridoxine non-responsive, treatment included a Met-restricted diet, supplemented with vitamin B6, betaine (150–250 mg/kg/day), folic acid (5 mg/day), and vitamin D and calcium for those with osteoporosis (P1 and P5).

One child (P7) was a 2-year-old girl who presented with developmental delay, microcephaly, and SE associated with febrile illness, and cranial magnetic resonance imaging (MRI) showed abnormal signals in both frontal lobes, possibly indicating poor myelination. A 24-h VEEG indicated bifrontal sharp and sharp slow waves. Plasma tHcy levels were elevated (85.7 μmol/L), with decreased Met level (6.8 μmol/L). Urine gas chromatography–mass spectrometry and blood tandem mass spectrometry results were normal. Treatment included anti-seizure medications, methyl cobalamin, calcium folinate, betaine, and vitamin B6. Over 15 months of follow-up, she achieved seizure freedom, developmental gains, and normalized tHcy levels. One patient (P8) was hospitalized because of cognitive dysfunction and urinary incontinence. After careful examination of the patient's medical history, the patient (P8) was hospitalized at the age of 6 years due to “viral encephalitis” and later left with epilepsy and unstable walking. He had elevated plasma tHcy (103.8 μmol/L) and urine methylmalonic acid levels (112.6 mg/g Cr) but decreased plasma Met level (4.5 μmol/L). Cranial MRI revealed brain atrophy. Treatment included hydroxycobalamin, oral betaine, L-carnitine, and folic acid. Over six months, his condition stabilized.

### 3.3 Genetic variation

The variations in the inherited HHcy-related genes identified in this study are summarized in Table 2. The cohort of eight patients demonstrated 11 variants in three genes, including nine previously reported pathogenic variants and 2 novel variants. All eight patients were compound heterozygotes. Seven *CBS* variants were identified in the CBS deficiency group. Among these, c.860del (p. Leu287ProfsTer7), a novel frameshift variant, was classified as pathogenic based on the American College of Medical Genetics (ACMG) criteria (PVS1 + PM2_S + PM3). The other six (c.374G>A, p. Arg125Gln; c.1006C>T, p. Arg336Cys; c.949A>G, p. Arg317Gly; c.346G>A, p. Gly116Arg; c.407T>C, p. Leu136Pro; c.442G>A, p. Gly148Arg) were previously reported as pathogenic missense variants. In patient P7, whole-exome sequencing identified compound heterozygous variants in the *MTHFR* gene: c.1632+2T>G (splicing) in exon 10 and c.1552C>T (p. Q518X, nonsense) in exon 9. The splicing variant c.1632+2T>G was previously reported and the other nonsense variant c.1552C>T was novel. Both were confirmed as likely pathogenic (PVS_M + PS3_S + PM2_S+PM1_S, and PVS + PM2_S) according to ACMG criteria. Sanger sequencing confirmed that her parents carried one heterozygous variant each. In patient P8, genetic testing revealed heterozygous variants in the *MMACHC* gene: c.482G >A (p. Arg161Gln) from his father, who was heterozygous, and c.609 G > A (p. Trp203^*^) from his heterozygous mother. Both variants had been previously reported as pathogenic.

**Table 2:** Genotypic features of eight Chinese patients with inherited HHcy.

**Patient/ sex**	**Gene**	**Transcript number**	**Exon/intron**	**Nucleotide change**	**Amino acid change**	**Source**
Pt1^a^/F	*CBS*	NM_000071.3	Exon5	c.374G>A	p. Arg125Gln	Paternal
	*CBS*	NM_000071.3	Exon11	c.1006C>T	p. Arg336Cys	Maternal
Pt2^a^/M	*CBS*	NM_000071.3	Exon5	c.374G>A	p. Arg125Gln	Paternal
	*CBS*	NM_000071.3	Exon11	c.1006C>T	p. Arg336Cys	Maternal
Pt3^b^/M	*CBS*	NM_000071.3	Exon10	c.949A>G	p. Arg317Gly	Paternal
	*CBS*	NM_000071.3	Exon5	c.346G>A	p. Gly116Arg	Maternal
Pt4^b^/F	*CBS*	NM_000071.3	Exon10	c.949A>G	p. Arg317Gly	Paternal
	*CBS*	NM_000071.3	Exon 5	c.346G>A	p. Gly116Arg	Maternal
Pt5/F	*CBS*	NM_000071.3	Exon5	c.374G>A	p. Arg125Gln	Paternal
	*CBS*	NM_000071.3	Exon10	c.860del	p. Leu287ProfsTer7	Maternal
Pt6/M	*CBS*	NM_000071.3	Exon3	c.407T>C	p. Leu136Pro	Paternal
	*CBS*	NM_000071.3	Exon3	c.442G>A	p. Gly148Arg	Maternal
Pt7/F	*MTHFR*	NM_005957	Exon10	c.1632+2T>G	Splicing	Paternal
	*MTHFR*	NM_001330358	Exon9	c.1552C>T	p. Q518X	Maternal
Pt8/M	*MMACHC*	NM_015506.3	Exon4	c.482G>A	p. Arg161Gln	Paternal
	*MMACHC*	NM_015506.3	Exon4	c.609G>A	p. Trp203^*^	Maternal

Pt, patient; F, female; M, male; HHcy, hyperhomocysteinemia. ^a, b^Siblings. ^*^Termination codon.

## 4 Discussion

Hcy, a sulfur-containing and non-essential amino acid, participates in a complex cycle involving Met and cysteine through a transsulfuration mechanism (3, 4). Hcy is converted to cysteine via transsulfuration or resynthesized back to Met via re-methylation, and Met undergoes recycling into Hcy via a methyl group donation reaction (3, 4). The transsulfuration pathway of Hcy requires the catalysis of vitamin B6-dependent CBS form cysteine (6). The re-methylation pathway is catalyzed by methyl-cbl-dependent methionine synthase, which requires 5- MTHF to provide the methyl group (6). Enzymatic deficiencies in these pathways can lead to abnormal Hcy metabolic cycle, and this can result in HHcy. HHcy is a rare neurometabolic syndrome posing significant diagnostic challenges in pediatric populations due to the diversity of its clinical manifestations and the overlap of abnormal biochemical metabolism (3). This retrospective review summarizes the clinical presentation, biochemical evaluation, variant spectrum, and treatment strategies for children/adolescents with inherited HHcy at a tertiary center in China.

The pathological factors of inherited HHcy include genetic variations and polymorphisms in key enzymes and coenzymes involved in metabolic pathways, such as CBS, MTHFR, MAT1A, and cbl metabolism-related genes (11). Diagnosis typically relies on medical history, genetic analysis, and detection of blood tHcy, Met, and urine organic acids (12). In this study, six patients (P1–P6) were diagnosed with CBS deficiency, one patient (P7) was confirmed to have MTHFR deficiency, and one patient (P8) was diagnosed with cblC deficiency. All eight patients had significantly elevated tHcy. Based on HHcy classification by plasma tHcy concentration (3, 4), seven patients (P1–P6, P8) had severe forms of HHcy (more than 100 μmol/L) of HHcy, and one patient (P7) had moderate form (50–100 umol/L) of HHcy. Moderate to severe HHcy leads to significant morbidity, impacting multiple organ systems (13). Seven patients (P1–P5, P7, and P8) had multisystem damage. One patient (P6) presented exclusively with ocular symptoms. The six patients with CBS deficiency experienced lens ectopia or myopia, presenting between 4 years and 6 years of age. Patient P7, diagnosed with MTHFR deficiency, displayed developmental delay and microcephaly at 1 year of age. One patient (P8) with cblC deficiency, presented with epilepsy and unstable walking. These findings are consistent with those of a previous study that showed that symptoms often emerge in early childhood, resulting in neurodevelopmental, skeletal, ocular, and vascular complications in inherited HHcy (14).

CBS deficiency is a rare autosomal recessive disease affecting 0.82 per 100,000 individuals globally (15). Without timely intervention, it can result in multisystemic disorders, including ocular diseases, intellectual disability, thromboembolic events, and skeletal abnormalities (7). In this group, eye disorders were the initial presentation in six patients with CBS deficiency, consistent with previous findings that congenital lens dislocation is the first symptom in most cases (16). We observed that all patients with CBS deficiency experienced lens ectopia and myopia. This is consistent with a previous finding that ectopia lentis was observed in 10 patients with classic homocystinuria (17). The exact mechanisms for ectopia lentis in classic homocystinuria due to CBS deficiency have not been fully understood. Rahman et al. (18) suggested that there was an alteration of self-interaction properties due to homocysteinylation of fibrillin-1 resulting from a reduction of disulfide-bonded C-terminal fibrillin-1 multimers, which could lead to weak zonules and progressive ectopia lentis. Furthermore, previous analysis indicated that elevated Hcy levels may interfere with the cross-linking of sulfhydryl groups in elastin (19, 20). The mechanism of high myopia may be due to the release of zonular tension from degeneration of zonular fibers that causes globe enlargement. Patients also develop early loss of accommodation due to zonular weakness (18). However, due to the condition's diverse manifestations and lack of specificity, diagnosis was often delayed. One child (P6) presented with isolated ocular disease and was treated with lensectomy with intraocular lens implantation. However, the treatment of ectopia lentis remains controversial. Surgical intervention is recommended for patients with anterior lens dislocation, with or without secondary acute glaucoma (19). However, surgical decisions must be made with caution in cases where the lens is dislocated into the vitreous cavity (21). Intellectual disability was observed in 83.3% of pediatric CBS deficiency cases in this study, likely due to the competitive inhibition of amino acid transport across the blood–brain barrier and elevated Met and Hcy in neurotransmitter synthesis (14). The Hcy level is an important risk factor for intracranial thrombosis (22). Up to 30% of patients with CBS deficiency experience vascular events before the age of 20, with half involving peripheral venous system thromboembolisms and up to 33% cerebrovascular accidents (14). Our data revealed that 50.0% of children/adolescents with CBS deficiency experienced CVST. The mean age of the patients with CVST was 12.7 ± 0.6 years (median, 13.0 years; range, 12.0–13.0 years). Wen et al. (16) reported that the prevalence of thrombotic complications in Chinese patients with CBS deficiency was 71.4% and that it increased with age. CVST was the most prominent clinical feature in the five HHcy patients with CBS deficiency. This phenomenon prompted us to pay more attention to CBS deficiency during routine thrombophilia screening in the Chinese population. CVST is a rare condition, responsible for 1–2% of strokes, with a prevalence of seven per million in children (23). Its clinical manifestations vary by venous occlusion site and include headaches, blurred vision, seizures, neurological deficits, and impaired consciousness (24). HHcy contributes to thrombosis through endothelial toxicity, smooth muscle cell proliferation, and intimal thickening (25). Three patients (P1, P3, and P5) with CVST were treated with mannitol, anticoagulants (low molecular weight heparin and warfarin), and homocysteine-lowering therapies. Apart from these, one patient (P3) was revealed to have extensive thrombosis in the intracranial venous sinus on computed tomography venography, experienced SE, and underwent percutaneous intracranial venous thrombectomy. After a median follow-up of 13 months, the patient showed greatly improved venous sinus thrombosis on cranial MRV, although residual abnormalities persisted. None of the patients experienced recurrent thrombosis, consistent with previous reports showing that anti-coagulant therapy and homocysteine reduction therapies effectively prevent recurrence (26). The standard treatment for CVST is anticoagulation therapy for the underlying cause. Intravascular thrombolysis using chemical thrombolysis, mechanical thrombectomy, or a combination of both should be considered in patients whose condition deteriorates despite adequate anticoagulation (27). Two children (P1, P3) with CBS deficiency had epilepsy, with plasma tHcy levels of 119.6 μmol/L and 227.2 μmol/L, respectively. This is consistent with a previous report showing that twenty percent of patients afflicted with CBS deficiency experience seizures that may develop into epilepsy when combined with elevated plasma tHcy concentrations of about 50–200 μmol/L (28). Epileptogenesis in HHcy may be attributed to elevated levels of cy-thiolactone, an Hcy metabolite (28). High levels of homocysteine and Met in urine and blood are characteristic biochemical findings of CBS deficiency. In our study, all six patients with CBS deficiency had significantly elevated plasma tHcy and Met levels, likely because CBS deficiency impairs the conversion of Hcy to CBS and leads to both Hcy and Met accumulation (6).

In this study, seven *CBS* variants were identified, including one novel frameshift variant (c.860del, p. Leu287ProfsTer7) from children and adolescents. According to the ACMG assessment criteria (29), c.860 del is a pathogenic variant (PVS1 + PM2_S + PM3). In addition, we identified two variants (c.949A>G, p. Arg317Gly and c.407 T>C, p. Leu136Pro) that have only been reported in Chinese populations (16, 25, 30). A previous study reported that two variant sites (c.949A>G and c.551T>C) appeared to be pyridoxal responsive, whereas the c.407 T>C variant was pyridoxal non-responsive (16). However, in our study, patient P6 with two variant sites (c.407T>C and c.442G>A) was pyridoxal non-responsive, while two patients (P3, P4) with two variant sites (c.949A>G and c.346G>A) also seemed to be pyridoxal non-responsive. We speculate that if a patient has a complex heterozygous *CBS* variant, pyridoxine responsiveness/non-responsiveness depends on the two variant sites. Notably, none of the hotspot variants previously reported in other regions were found. Chinese individuals may have *CBS* variant spectra that differ from those of other populations (16, 25, 31). The results of studies on other ethnic groups may not be directly applicable to the Chinese population; therefore, additional studies with larger patient cohorts are required to investigate the spectrum of CBS variants in Chinese patients. Met restriction, high dosage of B6, folate vitamins, and betaine in the diet, as well as cysteine supplementation, are the main treatment approaches. As key methyl donors, folate and betaine transmit methyl groups to Hcy to form Met, leading to a reduction in Hcy concentration through one-carbon metabolic pathways. Vitamin B6 is a cofactor for metabolic enzymes in a one-carbon cycle (32). B vitamins are widely used as nutritional supplements to reduce Hcy concentrations (33).

In this study, one patient (P7) was diagnosed with MTHFR deficiency, characterized by elevated plasma tHHcy levels and reduced Met concentrations. MTHFR disrupts folate acid metabolism, resulting in methyl tetrahydrofolate deficiency, HHcy, and hypomethioninemia. This autosomal recessive disorder is caused by a variant in *MTHFR*, which encodes MTHFR (4). Low Met results in decreased S-adenosylmethionine, that is vital for various methylation reactions (14). Variants in MTHFR would cause re-methylation disorders (4). Characteristics of the most prominently clinical signs for re-methylation defects are the developmental and neurocognitive impairment (3). MTHFR deficiency primarily presents in early childhood but can potentially present at any age with a high prevalence of cognitive impairment. Older infants and children frequently manifest with seizures, cognitive impairment, and acquired microcephaly (33). This aligns with the findings of our study, where we reported that one patient (P7) was diagnosed with MTHFR deficiency, characterized by developmental delay, SE, and microcephaly. The patient (P7) with MTHFR deficiency was diagnosed at 2 years of age. Two different variants (c.1632+2T>G, splicing, and c.1552C>T, p. Q518X) were defined, one of which was novel (c.1552C>T, p. Q518X). Family analysis revealed that the parents were carriers of a single variant. According to ACMG assessment criteria (29), c.1632+2T>G and c.1552C>T are both likely pathogenic variants (PVS_M + PS3_S + PM2_S+PM1_S, and PVS + PM2_S). The most frequent *MTHFR* variants in Chinese individuals are c.677C>T and c.1298A>T (3). Neither the p c.677C>T nor the c.1298A>T variants were detected in our cohort. The likely pathogenic and novel variant (c.1552C>T, p. Q518X) was detected in our cohort, which enriched the *MTHFR* variation spectrum. Treatment included anti-seizure medications, methylcobalamin, calcium folinate, betaine, and vitamin B6. On follow-up for 15 months, the patient became seizure-free with EEG normalization and had significant developmental gains and normalization of plasma tHcy levels. These findings suggest that early diagnosis and treatment are usually associated with good neurological outcomes in patients with MTHFR deficiencies. Yoganathan et al. reported improved outcomes with early intervention in MTHFR deficiency (6). However, additional studies with larger cohorts are needed to confirm these findings.

Methylcobalamin is essential for the cytosolic conversion of Hcy to Met, whereas adenosyl cobalamin is required for the intramitochondrial conversion of methylmalonyl-CoA to succinyl-CoA (6). Inborn errors in cobalamin metabolism can affect absorption, transportation, and genetic defects in intracellular cobalamin metabolism (4). The most common is a cblC deficiency caused by *MMACHC* variant, which has an autosomal recessive inheritance. Its main biological markers are HHcy, methylmalonic aciduria, and low/normal serum Met (33). These are caused by a decreased intracellular production of adenosylcobalamin and methylcobalamin, cofactors for the methylmalonyl-CoA mutase and methionine synthase enzymes. The deficient activity of these enzymes causes an elevation of methylmalonic acid (MMA) and Hcy (34, 35). Patient P8 had cblC with manifestations of epilepsy, unstable walking, cognitive dysfunction, and urinary incontinence. The *MMACHC* gene variants were compound heterozygous (c.482G>A, p. Arg161Gln; c.609G>A, p. Trp203^*^) in the patient (P8). In European patients, c.394C>T and c.482G>A are more common in late-onset cblC defects, whereas c.331C>T and c.271dupA are more common in the early-onset infantile type (36). However, the most common variants in Chinese patients with late-onset cblC deficiency were c.482G>A and c.609G>A (37). Acute decompensation should be managed using supportive therapy with betaine, parenteral hydroxocobalamin, carnitine, and folate. Long-term management should include administration of betaine, parenteral hydroxycobalamin, carnitine, and folate (33). Delayed diagnosis leads to severe irreversible neurological symptoms.

Hereditary HHcy is a group of inherited disorders of homocysteine metabolism, in which the pathological factors of hereditary HHcy include genetic variations and polymorphisms of key enzymes and coenzymes involved in metabolic pathways, such as CBS, MTHFR, and Cbl metabolism-related genes (3, 11). We retrospectively analyzed the data from eight children diagnosed with hereditary HHcy, and we summarized the biochemical characteristics, clinical manifestations, genetic variation, individualized treatment, and prognosis. Our results are consistent with previous findings that the main clinical manifestations of CBS deficiency include myopia, lens dislocation, Marfanoid morphotype, CVST, and intellectual disability, and psychiatric symptoms (14, 28). MTHFR deficiency can cause epilepsy, cognitive impairment, abnormal behavior, and microcephaly (33). CblC is the most common types of methylmalonic acidemia combined with homocysteinemia (3). The treatment approaches for different forms of HHcy vary. The initial treatment of CBS deficiency involves a therapeutic trial to determine vitamin B6 sensitivity, with one-third of patients responding positively. A low-protein diet, essential amino acid supplementation devoid of Met, and folate and vitamin B12 supplementation support Hcy re-methylation. Betaine supplementation, promoting re-methylation through alternative pathways, is considered. In cases resistant to vitamin B6, strict low-protein diets, Met-free essential amino acid formulas, and betaine administration are options (14). Guidelines for diagnosis and management of the cobalamin-related re-methylation disorders and MTHFR deficiency strongly recommend to initiate treatment with parenteral hydroxocobalamin without delay in any suspected re-methylation disorder, and betaine treatment in individuals with MTHFR deficiency, as these approaches have been confirmed to improve survival and reduce the incidence of severe complications when provided early (33). Additionally, our results failed to demonstrate or exclude a beneficial or detrimental effect of folic and/or folinic acid as adjunctive therapy in patients with cblC disease and other cobalamin-related re-methylation disorders (33). Unlike CBS deficiency, re-methylation disorders do not necessitate protein restriction, emphasizing the importance of maintaining normal Met levels. Therefore, individualized precision therapy, that incorporates dietary modifications and targeted supplementation is essential for optimizing outcomes in patients with HHcy (4).

In 2021, a retrospective analysis of healthcare claims in the United States between patients with diagnosed HHcy, and diagnosed phenylketonuria (PKU) reported that the highest proportion of diagnosed PKU patients was observed in the youngest age group (0–11 years), while the prevalence of diagnosed HHcy among younger patients was dramatically lower than that among older individuals. These data suggested that that infants with PKU are being diagnosed through effective universal newborn screening, while newborn screening failed to capture the vast majority of HHcy cases, often leaving patients without a diagnosis until late adulthood when they presented with serious symptoms or comorbid conditions indicative of HHcy (4). Inherited HHcy is a lifelong disorder caused by inborn errors of amino acid metabolism. Ideally, patients with inherited HHcy should be diagnosed as early as possible to minimize the symptoms and comorbidities associated with HHcy, and thus, further developments to newborn screening and detection in the pediatric population may be warranted. In China, Li et al. (3) retrospectively analyzed data from 84,722 newborns screened for hereditary HHcy over four years, and they suggested that the genetic variation spectrum could be determined using targeted gene panels for genetic screening in newborns or pregnant women. Additionally, testing of Hcy, MMA, and 2-methylcitric acid has typically been used as a second-tier marker in some locations such as Europe and Hangzhou, China to improve screening performance and increase specificity (38, 39). Therefore, early and effective screening, differential diagnosis, and individualized treatment of HHcy supported good prognosis in children with HHcy and reduced the burden of this disease among affected patients.

## 5 Conclusions

The identification of two novel variants enriches the understanding of the genetic spectrum of HHcy and provides a foundation for tailored genetic counseling and personalized treatment approaches. Inherited HHcy is a treatable, age-related condition, but its heterogeneous clinical presentations during infancy and childhood pose diagnostic challenges. Early and accurate etiologic diagnosis remains critical for initiating targeted therapies, improving outcomes, and preventing irreversible complications. The combination of metabolite screening and genetic testing can achieve rapid diagnosis and typing. This study contributes valuable data for the management of children/adolescents with inherited HHcy, particularly within the Chinese population, and emphasizes the need for continued research to refine diagnostic and therapeutic strategies.

## Data Availability

The original contributions presented in the study are included in the article/supplementary material, further inquiries can be directed to the corresponding author.
